# Effects of Simvastatin on Pharmacokinetics and Anticoagulant Effects of Dabigatran in Healthy Subjects

**DOI:** 10.3390/ph16030364

**Published:** 2023-02-27

**Authors:** Hyewon Chung, Jong-Min Kim, Jin-Woo Park, Jihyeon Noh, Kyoung-Ah Kim, Ji-Young Park

**Affiliations:** 1 Department of Clinical Pharmacology and Toxicology, Guro Hospital, Korea University College of Medicine, Seoul 08308, Republic of Korea; 2 Department of Clinical Pharmacology and Toxicology, Anam Hospital, Korea University College of Medicine, Seoul 02841, Republic of Korea; 3 Department of Neurology, Anam Hospital, Korea University College of Medicine, Seoul 02841, Republic of Korea

**Keywords:** dabigatran, simvastatin, interaction, pharmacokinetics

## Abstract

Higher risk of major hemorrhage associated with concomitant use of dabigatran and simvastatin compared to other statins was previously reported with a suggestion of P-glycoprotein-mediated interaction. The aim of this study was to evaluate the effects of simvastatin on pharmacokinetics and anticoagulant effects of dabigatran, a direct oral anticoagulant. A total of 12 healthy subjects were enrolled in an open-label, two-period, single sequence study. Subjects were given 150 mg of dabigatran etexilate followed by 40 mg of once-daily simvastatin for seven days. Dabigatran etexilate was administered with simvastatin on the seventh day of simvastatin administration. Blood samples for pharmacokinetic and pharmacodynamic analyses were obtained until 24 h post-dose of dabigatran etexilate with or without co-administration of simvastatin. Pharmacokinetic parameters were derived from noncompartmental analysis for dabigatran etexilate, dabigatran, and dabigatran acylglucuronide. When simvastatin was co-administered, geometric mean ratios of area under time-concentration curves for dabigatran etexilate, dabigatran, and dabigatran acylglucuronide were 1.47, 1.21, and 1.57, respectively, compared to when dabigatran etexilate was administered alone. Thrombin generation assay and coagulation assay showed similar profiles between before and after co-administration of simvastatin. This study provides evidence that simvastatin treatment plays a minor role in modulating pharmacokinetics and anticoagulant effects of dabigatran etexilate.

## 1. Introduction

Dabigatran, one of the novel oral anticoagulants, is a direct thrombin inhibitor [1]. It is used for the prevention and treatment of deep venous thrombosis and pulmonary embolism as well as for reducing risks of stroke and systemic embolism in patients with non-valvular atrial fibrillation [2]. Dabigatran etexilate is administered to patients as a prodrug since dabigatran is not orally available [3]. Its absolute bioavailability has been reported to be 7% [4]. After absorption, it undergoes two sequential metabolisms to form dabigatran and its glucuronide form, which are active metabolites pharmacologically. Carboxyesterase (CES1) is involved in the disposition of dabigatran etexilate [5]. It is rapidly absorbed and metabolized to dabigatran reaching maximum concentration (C_max_) at about 1.25–1.5 h after a single dose administration in healthy subjects [6]. Dabigatran is further metabolized to dabigatran acylglucuronide by uridine 5-diphospho (UDP)-glucuronosyltransferase (UGT) in the liver. Glucuronidation of the carboxylate moiety is the major human metabolic pathway of dabigatran and UGT2B15 is the major isoform involved in its glucuronidation [7]. Plasma protein binding of dabigatran is 35%, and 85% of the intravenous dabigatran is excreted via urine [4].

Statins are 3-hydroxy-3-methylglutaryl coenzyme A (HMG-CoA) reductase inhibitors that can lower cholesterol and reduce cardiovascular complications or mortality [8]. Statins can also be co-prescribed for patients taking dabigatran. Patients with atrial fibrillation with asymptomatic atherosclerosis should be treated with a statin and an oral anticoagulant [9]. Two studies including more than 90,000 patients using direct oral anticoagulant have reported a concomitant use of atorvastatin in 31.5% and 27.6% of patients [10,11].

Statins have a direct antithrombotic effect. They might interact with other antithrombotic drugs pharmacodynamically. It is known that co-prescription of a statin and warfarin can increase the risk of bleeding in patients [12,13]. Similarly, a case-control study has reported an association between major hemorrhage after use of dabigatran and co-administration with simvastatin [14]. However, reports are controversial since another clinical study has shown that the use of statin can reduce the risk of bleeding complication in patients taking dabigatran [15].

Simvastatin is a lactone form of prodrug for simvastatin acid, the active substance [16]. Time to reach C_max_ (T_max_) is about 1.8 h and 4.2 h for simvastatin and simvastatin acid, respectively [17]. It is extensively bound to plasma proteins and is primarily metabolized by CYP3A4 and excreted via bile [18]. It has a relatively short elimination half-life of about 2.8 h and 4.4 h for simvastatin and simvastatin acid, respectively [17]. Both dabigatran etexilate and simvastatin are substrates of P-glycoprotein (P-gp). Their competitive activities for P-gp might affect pharmacokinetics of dabigatran followed by bleeding complications. A clinical study has shown that co-administration of verapamil, a known P-gp inhibitor, with dabigatran etexilate can increase systemic exposure of dabigatran by 179%, indicating that dabigatran is a substrate of P-gp [19].

In addition to P-gp, there is also a possibility of simvastatin affecting pharmacokinetics of dabigatran via CES1 metabolism. Simvastatin shows inhibitory effects on imidapril hydrolase activities by recombinant CES1A1 and human liver microsomes [20]. Another in vitro study has reported that simvastatin can inhibit CES1-mediated hydrolysis of clopidogrel [21]. Therefore, simvastatin might inhibit the metabolism of dabigatran etexilate into dabigatran, leading to reduction of dabigatran levels and reduced anticoagulant effects.

Based on the wide use of statin and its potential concomitant use with dabigatran, pharmacokinetic interaction studies between atorvastatin and dabigatran in healthy volunteers have been conducted. No influence of atorvastatin on pharmacokinetics or pharmacodynamics of dabigatran has been reported [22]. However, no clinical studies evaluating pharmacokinetic interactions between dabigatran and simvastatin, which share metabolizing enzyme and drug transporter with each other, have been reported yet. Therefore, the objective of this study was to explore the effects of simvastatin on pharmacokinetics and anticoagulant effects of dabigatran in healthy subjects.

## 2. Results

### 2.1. Subjects

A total of 12 subjects were enrolled for this study. All 12 subjects were administered with dabigatran etexilate alone followed by 7 days of simvastatin administration where dabigatran etexilate was co-administered at the seventh day of simvastatin administration. No subject was dropped out and all 12 subjects completed the study. All subjects were males and their mean age, height, weight, and body mass index (BMI) were 25.8 years, 174 cm, 71.6 kg, and 23.6 kg/m^2^, respectively. With the exception of one subject experiencing mild pharyngitis, which resolved, there were no adverse events or clinically significant changes in vital signs, clinical laboratory tests, or electrocardiogram.

### 2.2. Effects of Simvastatin on Dabigatran Pharmacokinetics

**Figure 1:**
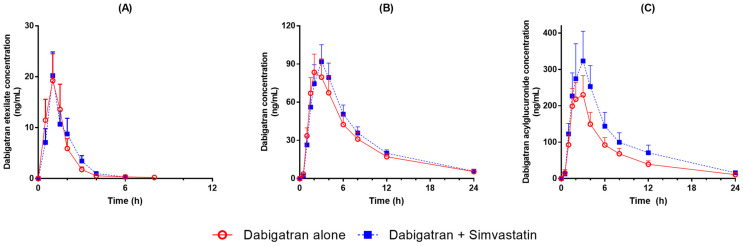
Mean plasma concentration profiles of (**A**) dabigatran etexilate, (**B**) dabigatran, and (**C**) dabigatran acylglucuronide after administration of 150 mg dabigatran etexilate with or without simvastatin.

Plasma concentrations of dabigatran etexilate, dabigatran, and dabigatran acylglucuronide were assessed before and after treatment with simvastatin. Average concentration profile of dabigatran etexilate was similar regardless of administration of simvastatin. However, those of dabigatran and dabigatran acylglucuronide tended to be higher after simvastatin co-administration compared to those without simvastatin co-administration (Figure 1). Pharmacokinetic characteristics of dabigatran etexilate, dabigatran, and dabigatran acylglucuronide did not exhibit any statistically significant difference between administration of dabigatran alone and co-administration with simvastatin (Table 1). When co-administrated with simvastatin, C_max_ values for dabigatran etexilate, dabigatran, and dabigatran acylglucuronide were 138%, 124%, and 163% of values when dabigatran was administrated alone, respectively. Similarly, area under the time–concentration curve from 0 h to the last measurable concentration (AUC_last_) values of dabigatran etexilate, dabigatran, and dabigatran acylglucuronide after co-administration with simvastatin were 147%, 121%, and 157% of values when they were administered alone, respectively. The mean AUC_last_ ratio of dabigatran acylglucuronide to dabigatran was 3.03 when dabigatran was administered alone. The ratio was higher (3.65) after co-administration with simvastatin (*p* = 0.0025).

### 2.3. Effects of Simvastatin on Dabigatran Anticoagulant Effects

**Figure 2:**
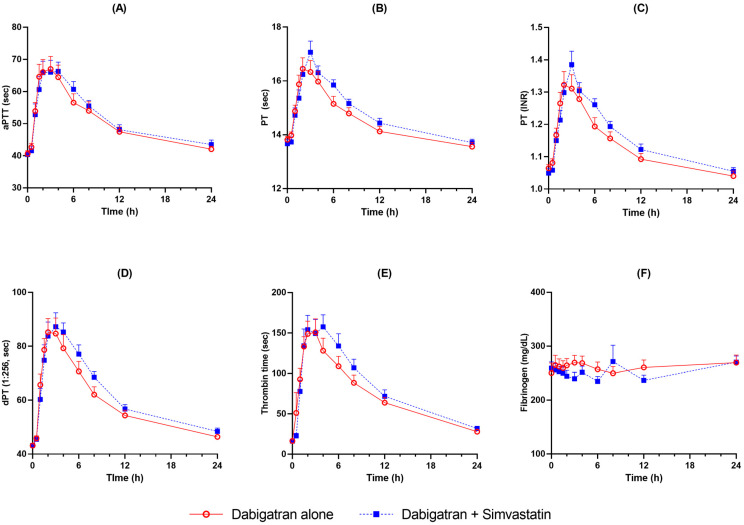
Coagulation assay profiles after administration of dabigatran etexilate alone and co-administration with simvastatin (activated partial prothrombin time (aPTT, (**A**)), prothrombin time (PT (s), (**B**)), prothrombin time (PT (INR), (**C**)), diluted PT (dPT, (**D**)), thrombin time (**E**), and fibrinogen (**F**)).

**Figure 3:**
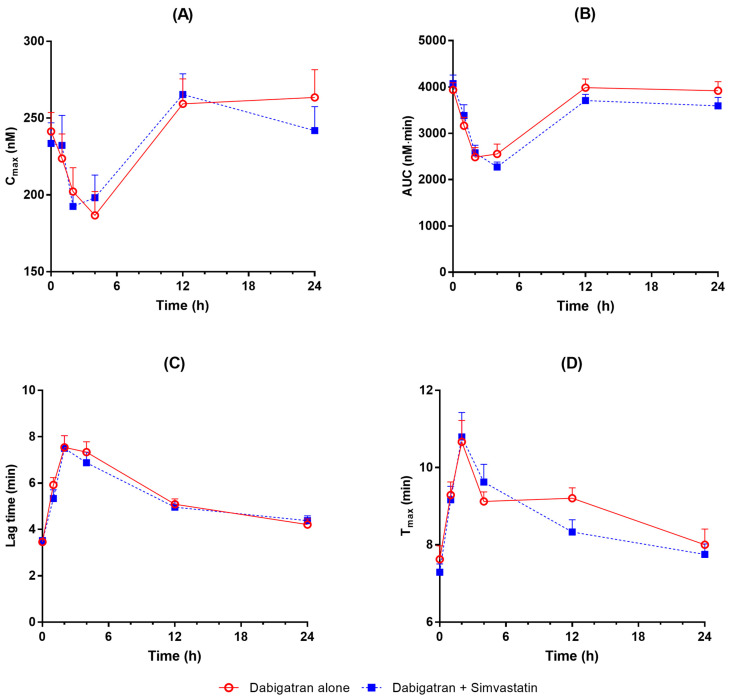
Thrombin generation assay profiles after administration of dabigatran alone and co-administration with simvastatin ((**A**) Peak thrombin concentration (C_max_), (**B**) endogenous thrombin potential (AUC), (**C**) lag time, and (**D**) time to C_max_ (T_max_)).

Baseline thrombin generation and coagulation assays measured before dabigatran administration were not different according to simvastatin administration (Table 2). Mean activated partial prothrombin time (aPTT), prothrombin time (PT), diluted PT (dPT), and thrombin time (TT) rose after the administration of dabigatran etexilate to reach their highest mean value at 2–4 h. Coagulation profiles were comparable between administration of dabigatran alone and co-administration with simvastatin (Figure 2). Area under the effect curve (AUEC) and maximum response (R_max_) values of pharmacodynamic parameters were not significantly different according to simvastatin co-administration except for AUEC of PT (Table 3). Thrombin generation assay (TGA) also showed similar profiles in terms of peak thrombin concentration (C_max_), endogenous thrombin potential (AUC), lag time, and T_max_ (Figure 3).

## 3. Discussion

In this study, we first explored the possibility of drug interaction by determining the effects of simvastatin on pharmacokinetics and anticoagulant effects of dabigatran in humans. The Canadian Drug Safety and Effectiveness Research Network team reported that the use of simvastatin or lovastatin is associated with a higher risk of major hemorrhage than other statins in patients taking dabigatran etexilate who require statin therapy [14]. It has been hypothesized that inhibition of intestinal P-gp by simvastatin increases dabigatran levels in blood, leading to potentiated anticoagulant effects of dabigatran since dabigatran is a substrate of P-gp. However, results of the present study did not show significant effects of simvastatin on dabigatran blood levels or anticoagulant effects.

AUC_last_ values of dabigatran etexilate, dabigatran, and dabigatran acylglucuronide after simvastatin co-administration increased 47%, 21%, and 57% compared to those after dabigatran administered alone, respectively. When the single dose pharmacokinetics of dabigatran etexilate is compared between healthy subjects and patients with renal impairment, the increase in exposure was 1.5 times in mild renal impairment patients [2]. Nevertheless, dose adjustment for these patients is not recommended. Therefore, pharmacokinetic changes observed in this study can be considered not clinically significant. Furthermore, anticoagulant effects including thrombin generation, PT, aPTT, dPT, and TT after dabigatran dosing were identical based on plasma concentration profiles over the time. Considering that dabigatran is a direct thrombin inhibitor, its anticoagulant effect would have moved the same way as blood dabigatran levels over the time [6]. Although AUEC of PT was statistically increased after simvastatin co-administration, it is difficult to establish clinical significance given that the R_max_ was similar. Furthermore, other coagulation tests such as aPTT and TT, which are reported to be more sensitive tests for dabigatran than PT, remained unchanged after the co-administration [23,24].

We could not fully understand the reason(s) why simvastatin clinically interacted with dabigatran etexilate and thereby increased the risk of bleeding in patients taking dabigatran in previous publications. Our findings suggest that pharmacokinetic interaction between dabigatran and simvastatin is not a key factor that increases the bleeding risk. As our study failed to show significant effects of simvastatin on pharmacokinetics and pharmacodynamics of dabigatran, it can be assumed that simvastatin itself has an anticoagulant effect in addition to dabigatran. Anticoagulant properties of statins reported in experimental and clinical studies involve decreased tissue factor expression that results in reduced thrombin generation and attenuation of pro-coagulant reactions as well as enhanced endothelial thrombomodulin expression [25]. However, when we assessed anticoagulant effects of simvastatin without dabigatran, there was no significant difference observed in thrombin generation or coagulation assays between before and after once daily administration of simvastatin for 7 days.

Simvastatin is a known inhibitor of P-gp transport [26,27]. It is also a substrate for P-gp [28]. Given that dabigatran etexilate is the substrate for P-gp, findings presented in this study suggest that P-gp might not be the main factor that causes clinical drug interaction between simvastatin and dabigatran, which causes hemorrhage in patients taking the two drugs concomitantly. A previous study has shown that verapamil, a potent inhibitor of P-gp, can elevate systemic exposure of total dabigatran (dabigatran + dabigatran acylglucuronide) by 54% compared to dabigatran alone [19]. In the case of fexofenadine, a substrate of P-gp, verapamil elevated systemic exposure of fexofenadine more than two-fold (261% for S-fexofenadine and 234% for R-fexofenadine) [29]. It implies that dabigatran might be a weak substrate of P-gp and that interaction with simvastatin plays a minor role in pharmacokinetic variability of dabigatran through P-gp transport.

Interestingly, average dabigatran acylglucuronide levels tended to be increased more than dabigatran levels after simvastatin treatment in this study. As mentioned above, dabigatran etexilate is a substrate of P-gp. It is mainly converted to its active metabolite, dabigatran, by CES1. Dabigatran is further metabolized to dabigatran acylglucuronide by UGT in the liver. Assuming that simvastatin acts as an inhibitor for P-gp-mediated transport or CES1 metabolism of dabigatran etexilate, systemic exposure of dabigatran and its acylglucuronide might have increased simultaneously. However, geometric mean ratios (GMRs) of pharmacokinetic parameters for dabigatran acylglucuronide was higher than those for dabigatran. Accordingly, mean AUC ratio of dabigatran acylglucuronide to dabigatran was significantly higher after co-administration with simvastatin. This implies that simvastatin might enhance metabolism of dabigatran to its acylglucuronide form. It has been reported that simvastatin acid undergoes glucuronidation in human microsomes and forms its lactone form [30]. In addition, efficacy of simvastatin has been found to be associated with single nucleotide polymorphisms in UGT1A1 [31]. However, there is no confirmatory evidence of whether simvastatin can induce UGT.

Our study has some limitations that should be acknowledged. First, we assessed the pharmacokinetics and anticoagulant effects of dabigatran in healthy subjects after a single dose of dabigatran, even though dabigatran is used for a long-term period. However, the objective of the present study was to assess the possibility of drug interaction between dabigatran and simvastatin. A single dose of dabigatran was justified based on linear pharmacokinetic characteristics with dose-proportional increases in pharmacokinetic parameters of dabigatran [6]. We first hypothesized that simvastatin was a perpetrator that could influence dabigatran pharmacokinetics. Therefore, simvastatin treatment was carried out for 7 days considering its long-term clinical use and to assess whether it exhibited anticoagulant effects itself. Second, a relatively small sample size showed a poor statistical power, which was inevitable for a complete pharmacokinetic study design with various anticoagulant assays. To exclude inter-subject variability and carry-over effect of simvastatin, single sequence crossover study design was adopted. The sample size was determined based on 15.18% of intra-subject variability in pharmacokinetic parameters of dabigatran assuming drop-out rate of 15% [32].

## 4. Materials and Methods

### 4.1. Subjects

All subjects provided signed informed consent prior to the screening procedure, which included vital signs, clinical laboratory tests, electrocardiogram, physical examination, and medical history. Healthy male subjects aged 19 to 50 with BMI of 18.5 to 29.9 kg/m^2^ were enrolled. Subjects who had clinically significant renal, gastrointestinal, hepatic, cardiovascular, respiratory, hematological, endocrinal, neurological, psychological, dermatological, or immune related medical condition or history were excluded. Other major exclusion criteria were: history of hypersensitivity to simvastatin or dabigatran, creatinine clearance less than 60 mL/min, aspartate aminotransferase or alanine aminotransferase exceeding two times of upper normal limit, and status of active bleeding or increased risk of bleeding, use of other anticoagulants or drugs known to have interaction with dabigatran or simvastatin prior to 2 weeks of study, participating in other clinical trials within 90 days of the study.

### 4.2. Study Design

An open-label, two-period, single sequence clinical study was conducted. After an overnight fast and drinking 200 mL of water one hour before drug administration, eligible subjects were administered with a capsule of dabigatran etexilate 150 mg (Pradaxa^®^) on the first day of the study. Blood samples for pharmacokinetic and pharmacodynamic analyses were obtained using EDTA tubes and citrate tubes, respectively. The samples were analyzed at pre-dose and at 0.5, 1, 1.5, 2, 3, 4, 6, 8, 12, and 24 h post-dose of dabigatran etexilate. Subjects remained at fasting stage until 4 h after the administration. From the second day of this study, subjects took simvastatin 40 mg (Zocor^®^) with 200 mL of water once daily for seven days at a fasting state. To ensure compliance, subjects visited the study site for every drug administration. On the last day of simvastatin administration, subjects were administered with dabigatran etexilate 150 mg one hour after simvastatin administration. Blood samples were obtained at the same time point scheduled as the first day of the study. Adverse events were collected during the entire study period. This study was conducted in accordance with the Declaration of Helsinki, good clinical practice, and local regulation. The study was approved by the Institutional Review Board at Korea University Guro Hospital (2018GR0336, date of approval: 11 October 2018) and registered at ClinicalTrials.gov (NCT03728101).

### 4.3. Determination of Dabigatran Etexilate, Dabigatran, and Dabigatran Acylglucuronide

Separate plasma samples were kept frozen below −70 ℃ until analysis. Plasma concentrations of dabigatran etexilate, dabigatran, and dabigatran acylglucuronide were quantified using a high-performance liquid chromatography (Nanospace series SI-2, Shiseido Co., Ltd., Tokyo, Japan) coupled with tandem mass spectrometry (API4000, AB SCIEX LLC, Framingham, MA, USA) method [33]. The method was validated in terms of selectivity, linearity, precision, accuracy, recovery, matrix effect, and stability prior to the analysis. In brief, 100 μL of plasma samples were deproteinized using 400 μL of acetonitrile and centrifuged at 13,000× *g* for 20 min. Dabigatran-d3 was used as an internal standard. The supernatants were evaporated and then dissolved in a 300 μL mobile phase consisting of acetonitrile and 10 mM ammonium formate (215:85, *v*/*v*). These samples were injected onto an Atlantis HILIC Silica™ column (2.1 mm × 150 mm, 3 μm, Waters, Milford, MA, USA) at a flow rate of 0.3 mL/min. The mass spectrometer equipped with an electrospray ionization source was operated in a positive ion mode. Selected ion pairs of *m*/*z* 629.464→290.100 for dabigatran etexilate, *m*/*z* 472.300→289.100 for dabigatran, *m*/*z* 648.382→289.100 for dabigatran acylglucuronide were detected. Calibration curves were linear over ranges of 0.2–500 ng/mL, 1–1000 ng/mL, and 1–1000 ng/mL for dabigatran etexilate, dabigatran, and dabigatran acylglucuronide, respectively. The accuracies of dabigatran etexilate, dabigatran, and dabigatran acylglucuronide were 95.84–109.44%, 99.4–103.42%, and 98.37–104.42%, respectively, while precisions of those were 3.84–9.79%, 1.07–8.76%, and 2.56–4.51%, respectively.

### 4.4. Anticoagulant Assay

Anticoagulant assays of aPTT, PT, dPT, TT, and fibrinogen were measured using STA^®^ Compact Max (Stago, Asnieres, France) according to the manufacturer’s instructions. Briefly, 100 μL of plasma sample and STA^®^ thrombin were mixed in a cuvette to measure TT. To measure PT, 100 μL of STA^®^ Neoplastine^®^ CI reagent was added to a cuvette with 50 μL of plasma. To measure aPTT, 50 μL of plasma sample was placed in a cuvette in the presence of STA^®^-PTT (50 μL) and 50 μL of 0.025 M CaCl_2_. For fibrinogen, 7.5 μL of plasma which was automatically diluted with Owren-Koller buffer was placed into a cuvette and then 50 μL of STA^®^-LIQID FIB was added.

TGA was performed using a Technothrombin^®^ TGA kit (Technoclone, Vienna, Austria) on a fully automated, computer-controlled microplate reader (Flexstation 3; Molecular Devices, San Jose, CA, USA) with a 360 nm excitation wavelength and 460 nm emission wavelength [34]. In brief, 30 μL of plasma samples were mixed with 25 μL of 400 μmol/L fluorogenic substrate (Z-Gly-Gly-Arg-AMC; Bachem, Bubendorf, Switzerland). The assay was initiated by dispensing 10 μL of 5 pmol/L recombinant human tissue factor lipidated with synthetic phospholipids and 10 μL of 15 mmol/L CaCl_2_. TGA results are expressed as C_max_, AUC, T_max_, and lag time.

### 4.5. Pharmacokinetic and Pharmacodynamic Analyses

Pharmacokinetic parameters of dabigatran etexilate, dabigatran, and dabigatran acylglucuronide were derived from noncompartmental analysis using Phoenix Winnonlin^®^ version 8.3 (Certara, Princeton, NJ, USA). Observed values from individual time-concentration profiles were used for C_max_ and T_max_. Area under the time-concentration curve (AUC) was calculated using linear-up log-down trapezoidal rule. The AUC from 0 h to infinity (AUC_inf_) was calculated as follows: AUC_inf_ = AUC + C_t_/k_e_, where C_t_ was the last plasma concentration and k_e_ was the elimination rate constant. The terminal elimination half-life (t_1/2_) was calculated as the natural logarithm of 2 divided by k_e_. Mean residence time (MRT) was calculated as area under the moment curve from time of dosing to the last measurable concentration divided by AUC_last_.

For pharmacodynamic assessment, Phoenix Winnonlin^®^ version 8.3 (Pharsight, Mountain View, CA, USA) was used to calculate AUEC with the linear trapezoidal method and R_max_ was directly determined from individual profiles.

### 4.6. Statistical Analysis

All statistical analyses were performed using SAS^®^ version 9.4 (SAS Institute Inc., Cary, NC, USA). Descriptive statistics were used to summarize demographic, pharmacokinetic, and pharmacodynamic data. A linear mixed effects model was used to compare C_max_, AUC_last_, and AUC_inf_ between two treatment periods. GMRs and their 90% confidence interval (CI) were derived from the model by applying the exponential function to the difference of least squares means between the two periods. Parameters before and after co-administration with simvastatin were compared using paired *t*-test, where *p*-values less than 0.05 were considered statistically significant.

## 5. Conclusions

In conclusion, results of the present study provide evidence that simvastatin treatment plays a minor role in modulating pharmacokinetics and anticoagulant effects of dabigatran etexilate in humans.

## Figures and Tables

**Table 1:** Pharmacokinetic parameters of dabigatran etexilate, dabigatran, and dabigatran glucuronide before and after co-administration of simvastatin.

	Parameter	Dabigatran Alone	Dabigatran Co-Administered with Simvastatin	GMR (90% CI)	*p*-Value
Dabigatran etexilate	AUC_inf_ (h∙ng/mL)	33.7 (26.0)	36.4 (18.5)	1.60 (0.89–2.88)	0.3847
	AUC_last_ (h∙ng/mL)	26.1 (23.6)	27.3 (16.4)	1.47 (0.87–2.48)	0.8242
	CL/F (L/h)	12,208.4 (16,447.7)	5235.1 (2729.4)	-	
	C_max_ (ng/mL)	24.5 (19.2)	25.1 (13.8)	1.38 (0.81–2.35)	0.9139
	t_1/2_ (h)	3.4 (5.8)	6.4 (13.6)	-	
	MRT (h)	1.9 (2.4)	2.2 (1.8)	-	
	T_max_ (h)	1 (0.5–1.5)	1 (0.5–2)	-	
Dabigatran	AUC_inf_ (h∙ng/mL)	663.9 (377.5)	740.2 (342.0)	1.19 (0.71–2.00)	0.6033
	AUC_last_ (h∙ng/mL)	617.1 (357.4)	688.2 (318.9)	1.21 (0.71–2.08)	0.6079
	CL/F (L/h)	362.3 (331.8)	275.8 (197.4)	-	
	C_max_ (ng/mL)	87.0 (51.4)	98.6 (47.5)	1.24 (0.72–2.12)	0.5601
	t_1/2_ (h)	6.1 (0.9)	6.2 (0.6)	-	
	MRT (h)	7.1 (0.9)	7.5 (0.7)	-	
	T_max_ (h)	2 (1–4)	3 (1.5–4)	-	
Dabigatran acylglucuronide	AUC_inf_ (h∙ng/mL)	1576.5 (1154.0)	2376.8 (2096.8)	1.56 (0.94–2.59)	0.2903
	AUC_last_ (h∙ng/mL)	1480.1 (1090.5)	2227.5 (1994.6)	1.57 (0.93–2.66)	0.3042
	CL/F (L/h)	176.8 (161.8)	97.1 (56.1)	-	
	C_max_ (ng/mL)	266.8 (187.1)	392.0 (290.2)	1.63 (0.94–2.80)	0.2885
	t_1/2_ (h)	6.4 (1.9)	6.0 (1.8)	-	
	MRT (h)	6.8 (0.9)	7.1 (0.8)	-	
	T_max_ (h)	2 (1.5–4)	2.5 (1–4)	-	
AUC_last_ ratio of dabigatran acylglucuronide to dabigatran		3.03 (2.42)	3.65 (2.54)	1.30 (1.15–1.46)	0.0025

Data are presented as mean (standard deviation) except for T_max_ which is presented as median (min–max). GMR: geometric mean ratio, CI: confidence interval, AUC_inf_: area under the time-concentration curve from 0 h to infinity, AUC_last_: area under the time-concentration curve from 0 h to the last measurable concentration, CL/F: apparent clearance, C_max_: maximum concentration, t_1/2_: terminal elimination half-life, MRT: mean residence time, T_max_: time to reach C_max._

**Table 2:** Baseline thrombin generation and coagulation assays measured before dabigatran etexilate administration.

		Dabigatran Alone	Dabigatran with Simvastatin	*p*-Value
Thrombin Generation Assay	C_max_ (nM)	241.3 (43.0)	233.6 (46.7)	0.5629
	AUC (nM∙min)	3937.1 (537.4)	4073.1 (642.2)	0.3383
	lag time (min)	3.5 (0.5)	3.5 (0.5)	0.3388
	T_max_ (min)	7.6 (1.2)	7.3 (0.8)	0.2072
Coagulation Assay	aPTT (s)	40.6 (3.3)	40.4 (3.3)	0.6502
	PT (s)	13.8 (0.4)	13.7 (0.6)	0.2068
	PT (INR)	1.1 (0.0)	1.0 (0.1)	0.1718
	dPT (s)	43.2 (1.7)	43.2 (1.8)	0.9726
	TT (s)	16.5 (1.2)	16.2 (0.9)	0.4361
	Fibrinogen (mg/dL)	250.3 (63.9)	259.3 (39.4)	0.5432

Data are presented as mean (standard deviation). C_max_: Peak thrombin concentration, AUC: endogenous thrombin potential, T_max_: time to C_max_, aPTT: activated partial prothrombin time, PT: prothrombin time, dPT: dilute PT, TT: thrombin time.

**Table 3:** Pharmacodynamic parameters of dabigatran before and after co-administration of simvastatin.

		Parameter	Dabigatran Alone	Dabigatran with Simvastatin	*p*-Value
Thrombin Generation	C_max_	AUEC (nM∙h)	5755.0 (1129.2)	5734.0 (993.9)	0.9369
		R_max_ (nM)	180.2 (48.8)	177.8 (35.8)	0.8684
	AUC	AUEC (nM∙min∙h)	84,994.8 (14,977.1)	79,286.4 (10,676.3)	0.0746
		R_max_ (nM∙min)	2423.6 (734.9)	2231.7 (326.0)	0.2944
	Lag time	AUEC (min∙h)	131.7 (20.8)	128.5 (20.1)	0.6835
		R_max_ (min)	7.8 (1.7)	8.0 (1.5)	0.7206
	T_max_	AUEC (min∙h)	214.8 (21.9)	207.0 (22.0)	0.2667
		R_max_ (min)	11.0 (1.6)	11.3 (1.7)	0.6767
Coagulation assay	aPTT	AUEC (s∙h)	1203.1 (153.1)	1235.7 (131.7)	0.4873
		R_max_ (s)	69.5 (13.8)	71.7 (12.2)	0.6408
	PT (sec)	AUEC (s∙h)	344.9 (14.3)	353.8 (12.3)	0.0331
		R_max_ (s)	16.6 (1.4)	17.2 (1.4)	0.3048
	PT (INR)	AUEC	26.9 (1.4)	27.7 (1.2)	0.0474
		R_max_	1.3 (0.1)	1.4 (0.1)	0.3038
	dPT (1:256)	AUEC (s∙h)	1404.8 (184.7)	1482.9 (144.9)	0.2977
		R_max_ (s)	87.5 (19.2)	92.5 (15.3)	0.5068
	TT	AUEC (s∙h)	1690.1 (535.9)	1976.2 (643.8)	0.3393
		R_max_ (s)	169.2 (74.0)	180.7 (50.4)	0.6841
	Fibrinogen	AUEC (h∙mg/dL)	6249.8 (1133.1)	6040.9 (758.3)	0.4101
		R_max_ (mg/dL)	288.0 (55.9)	309.3 (96.0)	0.4907

Data are presented as mean (standard deviation). C_max_: peak thrombin concentration, AUC: endogenous thrombin potential, T_max_: time to C_max_, aPTT:,activated partial prothrombin time, PT: prothrombin time, dPT: dilute PT, TT: thrombin time, AUEC: area under the effect curve, R_max_: maximum response.

## Data Availability

Data presented in this study are available from the corresponding author upon reasonable request. The data are not publicly available due to the privacy issue of subjects.

## References

[1] Wienen W., Stassen J.M., Priepke H., Ries U.J., Hauel N. (2007). In-vitro profile and ex-vivo anticoagulant activity of the direct thrombin inhibitor dabigatran and its orally active prodrug, dabigatran etexilate. Thromb. Haemost..

[2] PRADAXA® Label. https://www.accessdata.fda.gov/drugsatfda_docs/label/2021/022512s041lbl.pdf.

[3] Stangier J. (2008). Clinical pharmacokinetics and pharmacodynamics of the oral direct thrombin inhibitor dabigatran etexilate. Clin. Pharmacokinet..

[4] Blech S., Ebner T., Ludwig-Schwellinger E., Stangier J., Roth W. (2008). The metabolism and disposition of the oral direct thrombin inhibitor, dabigatran, in humans. Drug Metab. Dispos. Biol. Fate Chem..

[5] Laizure S.C., Parker R.B., Herring V.L., Hu Z.Y. (2014). Identification of carboxylesterase-dependent dabigatran etexilate hydrolysis. Drug Metab. Dispos. Biol. Fate Chem..

[6] Stangier J., Rathgen K., Stahle H., Gansser D., Roth W. (2007). The pharmacokinetics, pharmacodynamics and tolerability of dabigatran etexilate, a new oral direct thrombin inhibitor, in healthy male subjects. Br. J. Clin. Pharmacol..

[7] Ebner T., Wagner K., Wienen W. (2010). Dabigatran acylglucuronide, the major human metabolite of dabigatran: In vitro formation, stability, and pharmacological activity. Drug Metab. Dispos. Biol. Fate Chem..

[8] Baigent C., Keech A., Kearney P.M., Blackwell L., Buck G., Pollicino C., Kirby A., Sourjina T., Peto R., Collins R. (2005). Efficacy and safety of cholesterol-lowering treatment: Prospective meta-analysis of data from 90,056 participants in 14 randomised trials of statins. Lancet.

[9] Steffel J., Collins R., Antz M., Cornu P., Desteghe L., Haeusler K.G., Oldgren J., Reinecke H., Roldan-Schilling V., Rowell N. (2021). 2021 European Heart Rhythm Association Practical Guide on the Use of Non-Vitamin K Antagonist Oral Anticoagulants in Patients with Atrial Fibrillation. Eur. Eur. Pacing Arrhythm. Card. Electrophysiol. J. Work. Groups Card. Pacing Arrhythm. Card. Cell. Electrophysiol. Eur. Soc. Cardiol..

[10] Chang S.H., Chou I.J., Yeh Y.H., Chiou M.J., Wen M.S., Kuo C.T., See L.C., Kuo C.F. (2017). Association Between Use of Non-Vitamin K Oral Anticoagulants With and Without Concurrent Medications and Risk of Major Bleeding in Nonvalvular Atrial Fibrillation. JAMA.

[11] Harskamp R.E., Himmelreich J.C.L., Wong G.W.M., Teichert M. (2021). Prescription patterns of direct oral anticoagulants and concomitant use of interacting medications in the Netherlands. Neth. Heart J. Mon. J. Neth. Soc. Cardiol. Neth. Heart Found..

[12] Douketis J.D., Melo M., Bell C.M., Mamdani M.M. (2007). Does statin therapy decrease the risk for bleeding in patients who are receiving warfarin?. Am. J. Med..

[13] Schelleman H., Bilker W.B., Brensinger C.M., Wan F., Yang Y.X., Hennessy S. (2010). Fibrate/Statin initiation in warfarin users and gastrointestinal bleeding risk. Am. J. Med..

[14] Antoniou T., Macdonald E.M., Yao Z., Hollands S., Gomes T., Tadrous M., Mamdani M.M., Juurlink D.N., Canadian Drug S. (2017). Association between statin use and ischemic stroke or major hemorrhage in patients taking dabigatran for atrial fibrillation. CMAJ Can. Med. Assoc. J. = J. l’Association Med. Can..

[15] Ho B.L., Lin Y.J., Lin S.F., Chou P.S., Chen C.F., Lin R.T., Hu H.H., Chao A.C. (2019). Statins and the risk of bleeding in patients taking dabigatran. Acta Neurol. Scand..

[16] Mauro V.F. (1993). Clinical pharmacokinetics and practical applications of simvastatin. Clin. Pharmacokinet..

[17] Najib N.M., Idkaidek N., Adel A., Admour I., Astigarraga R.E., Nucci G.D., Alam S.M., Dham R. (2003). Pharmacokinetics and bioequivalence evaluation of two simvastatin 40 mg tablets (Simvast and Zocor) in healthy human volunteers. Biopharm. Drug Dispos..

[18] Schachter M. (2005). Chemical, pharmacokinetic and pharmacodynamic properties of statins: An update. Fundam. Clin. Pharmacol..

[19] Hartter S., Sennewald R., Nehmiz G., Reilly P. (2013). Oral bioavailability of dabigatran etexilate (Pradaxa((R))) after co-medication with verapamil in healthy subjects. Br. J. Clin. Pharmacol..

[20] Fukami T., Takahashi S., Nakagawa N., Maruichi T., Nakajima M., Yokoi T. (2010). In vitro evaluation of inhibitory effects of antidiabetic and antihyperlipidemic drugs on human carboxylesterase activities. Drug Metab. Dispos. Biol. Fate Chem..

[21] Wang X., Zhu H.J., Markowitz J.S. (2015). Carboxylesterase 1-mediated drug-drug interactions between clopidogrel and simvastatin. Biol. Pharm. Bull..

[22] Stangier J., Rathgen K., Stahle H., Reseski K., Kornicke T., Roth W. (2009). Coadministration of dabigatran etexilate and atorvastatin: Assessment of potential impact on pharmacokinetics and pharmacodynamics. Am. J. Cardiovasc. Drugs Drugs Devices Other Interv..

[23] Van Ryn J., Stangier J., Haertter S., Liesenfeld K.H., Wienen W., Feuring M., Clemens A. (2010). Dabigatran etexilate--a novel, reversible, oral direct thrombin inhibitor: Interpretation of coagulation assays and reversal of anticoagulant activity. Thromb. Haemost..

[24] Dager W.E., Gosselin R.C., Kitchen S., Dwyre D. (2012). Dabigatran effects on the international normalized ratio, activated partial thromboplastin time, thrombin time, and fibrinogen: A multicenter, in vitro study. Ann. Pharmacother..

[25] Undas A., Brummel-Ziedins K.E., Mann K.G. (2014). Anticoagulant effects of statins and their clinical implications. Thromb. Haemost..

[26] Bogman K., Peyer A.K., Torok M., Kusters E., Drewe J. (2001). HMG-CoA reductase inhibitors and P-glycoprotein modulation. Br. J. Pharmacol..

[27] Wang E., Casciano C.N., Clement R.P., Johnson W.W. (2001). HMG-CoA reductase inhibitors (statins) characterized as direct inhibitors of P-glycoprotein. Pharm. Res..

[28] Hochman J.H., Pudvah N., Qiu J., Yamazaki M., Tang C., Lin J.H., Prueksaritanont T. (2004). Interactions of human P-glycoprotein with simvastatin, simvastatin acid, and atorvastatin. Pharm. Res..

[29] Sakugawa T., Miura M., Hokama N., Suzuki T., Tateishi T., Uno T. (2009). Enantioselective disposition of fexofenadine with the P-glycoprotein inhibitor verapamil. Br. J. Clin. Pharmacol..

[30] Prueksaritanont T., Subramanian R., Fang X., Ma B., Qiu Y., Lin J.H., Pearson P.G., Baillie T.A. (2002). Glucuronidation of statins in animals and humans: A novel mechanism of statin lactonization. Drug Metab. Dispos. Biol. Fate Chem..

[31] Iwuchukwu O.F., Feng Q., Wei W.Q., Jiang L., Jiang M., Xu H., Denny J.C., Wilke R.A., Krauss R.M., Roden D.M. (2014). Genetic variation in the UGT1A locus is associated with simvastatin efficacy in a clinical practice setting. Pharmacogenomics.

[32] Stangier J., Stahle H., Rathgen K., Fuhr R. (2008). Pharmacokinetics and pharmacodynamics of the direct oral thrombin inhibitor dabigatran in healthy elderly subjects. Clin. Pharmacokinet..

[33] Park I.H., Park J.W., Chung H., Kim J.M., Lee S., Kim K.A., Park J.Y. (2021). Development and validation of LC-MS/MS method for simultaneous determination of dabigatran etexilate and its active metabolites in human plasma, and its application in a pharmacokinetic study. J. Pharm. Biomed. Anal..

[34] Kim J.M., Noh J., Park J.W., Chung H., Kim K.A., Park S.B., Lee J.S., Park J.Y. (2022). Dabigatran Acylglucuronide, the Major Metabolite of Dabigatran, Shows a Weaker Anticoagulant Effect than Dabigatran. Pharmaceutics.

